# Functional Profiles of Fish Gut Microbiota Associated With Feeding Habits in the Midstream of the Jialing River Under Cascade Channelization

**DOI:** 10.1002/ece3.74414

**Published:** 2026-10-02

**Authors:** Mengying Hu, Chang Chen, Yuan Feng, Liang Liu, Xiaopeng Tang, Heran Wang, Fubin Zhang

**Affiliations:** ^1^ College of Environmental Science and Engineering China West Normal University Nanchong China; ^2^ Ministry of Education Key Laboratory of Southwest China Wildlife Resources Conservation (China West Normal University) Nanchong China

**Keywords:** cascade channelization, feeding habit, fish gut microbiota, functional prediction, Jialing River, metabolic pathways

## Abstract

To examine the influence of diet on fish gut microbiota in channelized rivers, we characterized the gut microbial communities of fish with different feeding habits in the midstream of the Jialing River. The main findings are as follows: (1) 16S rRNA gene amplicon sequencing identified Firmicutes and Proteobacteria as the dominant phyla, with their relative abundances differing significantly among fish of distinct feeding habits. Firmicutes exhibited the highest relative abundance in carnivorous fish (93.41%), followed by omnivorous (53.39%) and herbivorous fish (29.3%). In contrast, Proteobacteria was most abundant in herbivorous fish (32.72%), with lower levels in omnivorous (22.43%) and carnivorous fish (4.81%). (2) The Kruskal–Wallis test indicated significant differences in the α‐diversity of gut microbial communities among fish with distinct feeding habits (*p* < 0.05), where herbivorous fish presented the highest α‐diversity, followed by omnivorous fish, with carnivorous fish exhibiting the lowest. PERMANOVA analysis showed that the feeding habits significantly influenced gut microbial community structure (Pseudo‐*F* = 3.85, *R* ^2^ = 0.18033, *p* < 0.05). (3) PICRUSt2 functional prediction analysis showed that omnivorous fish in the midstream of the Jialing River had a higher relative abundance of pathways related to microbial metabolism in diverse environments (4.792% ± 0.221%). In comparison, herbivorous fish had a higher relative abundance of pathways related to carbon fixation pathways in prokaryotes (0.927% ± 0.091%). In contrast, carnivorous fish not only had a higher relative abundance of the cysteine and methionine metabolism pathway (1.026% ± 0.051%) but also showed higher relative abundances of pathways related to carbohydrate utilization, including starch and sucrose metabolism (0.768% ± 0.031%) and the phosphotransferase system (PTS) (0.461% ± 0.019%), which may be associated with habitat homogenization following channelization. These findings provide a reference for habitat restoration in channelized rivers and the conservation of fish resources.

## Graphical abstract

This study investigates how feeding habits shape the gut microbiota of fish inhabiting the channelized middle reaches of the Jialing River. Using 16S rRNA gene amplicon sequencing and PICRUSt2 analysis, we compared the gut microbial communities of carnivorous, omnivorous, and herbivorous fish. Our results demonstrate that feeding habits are a key determinant of microbial community structure, with distinct compositional profiles and diversity patterns observed across dietary groups. Furthermore, functional prediction revealed differential enrichment of specific metabolic pathways among the groups. Notably, carnivorous fish exhibited higher relative abundances of pathways related to carbohydrate utilization, a finding that may reflect an adaptive response to altered resource availability in homogenized, channelized habitats.

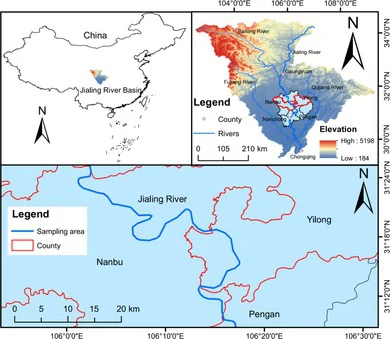

## 1 Introduction

The gut microbiota of fish plays a crucial role in host nutrient metabolism and environmental adaptation, providing insights into the mechanisms by which hosts adapt to their surroundings (Wu, Pan, et al. 2024, Wu, Xiao, et al. 2024, Ou et al. 2021). This field represents a prominent research focus in aquatic ecology (Fu et al. 2017). Recent advancements in molecular ecology have significantly propelled the study of fish gut microbiota (Leroux et al. 2023).

Diet plays an important role in influencing fish gut microbial community structure (Pérez‐Pascual et al. 2020). Typically, significant interspecific differences are observed in the gut microbiota among sympatric fish species with distinct feeding habits (Liang et al. 2024; Li et al. 2018), whereas fish sharing similar dietary niches tend to harbor more comparable microbial communities. These patterns are likely associated with variations in feeding behavior, dietary composition, and the resultant gut physicochemical environment (Long et al. 2023; Chen et al. 2022). Furthermore, habitat characteristics have a substantial influence on the assembly of fish gut microbiota. Conspecific fish inhabiting different geographical locations or habitat types often exhibit marked divergence in their gut microbial community structure (Gu et al. 2021; Viver et al. 2023). This indicates that, alongside diet, habitat variation is a critical factor influencing the configuration of the fish gut microbiota (Kim et al. 2021; Bi et al. 2021). Such differences primarily stem from distinct combinations of environmental factors—such as water temperature, salinity, pH, and nutrient concentrations—as well as from variations in prey availability and the resultant disparities in environmental microbial communities (Li et al. 2023; Kanika et al. 2025).

At the level of metabolic function, the enrichment of metabolic pathways in the fish gut microbiota is closely associated with feeding habits (Li et al. 2025; Deng et al. 2024). Carnivorous fish, consuming high‐protein diets, often harbor gut microbial communities enriched in pathways related to amino acid metabolism, such as cysteine and methionine metabolism, to facilitate efficient protein utilization (Mujar et al. 2025; Louvado et al. 2024). Omnivorous fish, with their diverse diets, typically exhibit higher microbial diversity. Consequently, broad‐spectrum metabolic pathways like microbial metabolism in diverse environments often show higher relative abundance (Boonanuntanasarn et al. 2018; Kumkhong et al. 2020). Herbivorous fish, primarily feeding on aquatic macrophytes, algae, and organic detritus, ingest diets typically rich in structurally complex carbohydrates (e.g., cellulose and hemicellulose) (Li et al. 2019). Accordingly, pathways related to carbon fixation, such as carbon fixation pathways in prokaryotes, are often present at relatively higher abundance in their intestines (Banik et al. 2025; Ji et al. 2025; Wei et al. 2024).

The Jialing River is the largest tributary of the Yangtze River basin by drainage area, spanning 1345 km in total length and approximately 160,000 km^2^ in catchment (Zhang et al. 2020). Its middle reaches, stretching 633 km from Zhaohua to Hechuan, have been fully channelized through the construction of 13 cascade hydropower dams (Wang et al. 2021; Deng et al. 2025). This cascade dam construction has substantially altered the riverine environment, leading to reduced flow velocity, habitat homogenization, and a shift in prey communities from lotic‐ to lentic‐adapted species (Yi et al. 2019; Jiang et al. 2023). Such pronounced anthropogenic disturbance may influence the composition and function of fish gut microbiota through mechanisms including altered food web structure and trophic cascades (Wang et al. 2023; Soomro et al. 2023). Compared to natural riverine and lacustrine ecosystems, the diet‐community structure–function relationship of fish gut microbiota in channelized rivers may be significantly modified (Zheng et al. 2024).

This study selected dominant species with different feeding habits from the channelized section of the midstream of the Jialing River as research subjects (Qin et al. 2023). Through 16S rRNA gene high‐throughput sequencing and PICRUSt2 functional prediction, it aims to address the following questions: (1) whether gut microbial community structure differs among fish with different feeding habits in the channelized river; (2) what are the characteristics of gut microbial diversity among fish with different feeding habits in the channelized river; and (3) whether PICRUSt2‐predicted metabolic functions differ among fish with different feeding habits, and whether these differences align with diet‐associated patterns reported in previous studies. By examining these aspects, this study seeks to provide new insights from a microbial ecology perspective into fish ecological adaptation in cascade‐channelized rivers, thereby offering a theoretical framework for fish resource conservation and habitat restoration.

## 2 Materials and Methods

### 2.1 Study Area and Sample Processing

The midstream of the Jialing River serves as a vital ecological corridor in the upper Yangtze River and is a key region for maintaining biodiversity (Zhou et al. 2020). The construction of cascade dams in this area has resulted in habitat homogenization and a significant reduction in hydrological connectivity, providing an ideal setting for studying the ecological adaptation of fish under channelized conditions (Figure 1).

**FIGURE 1:**
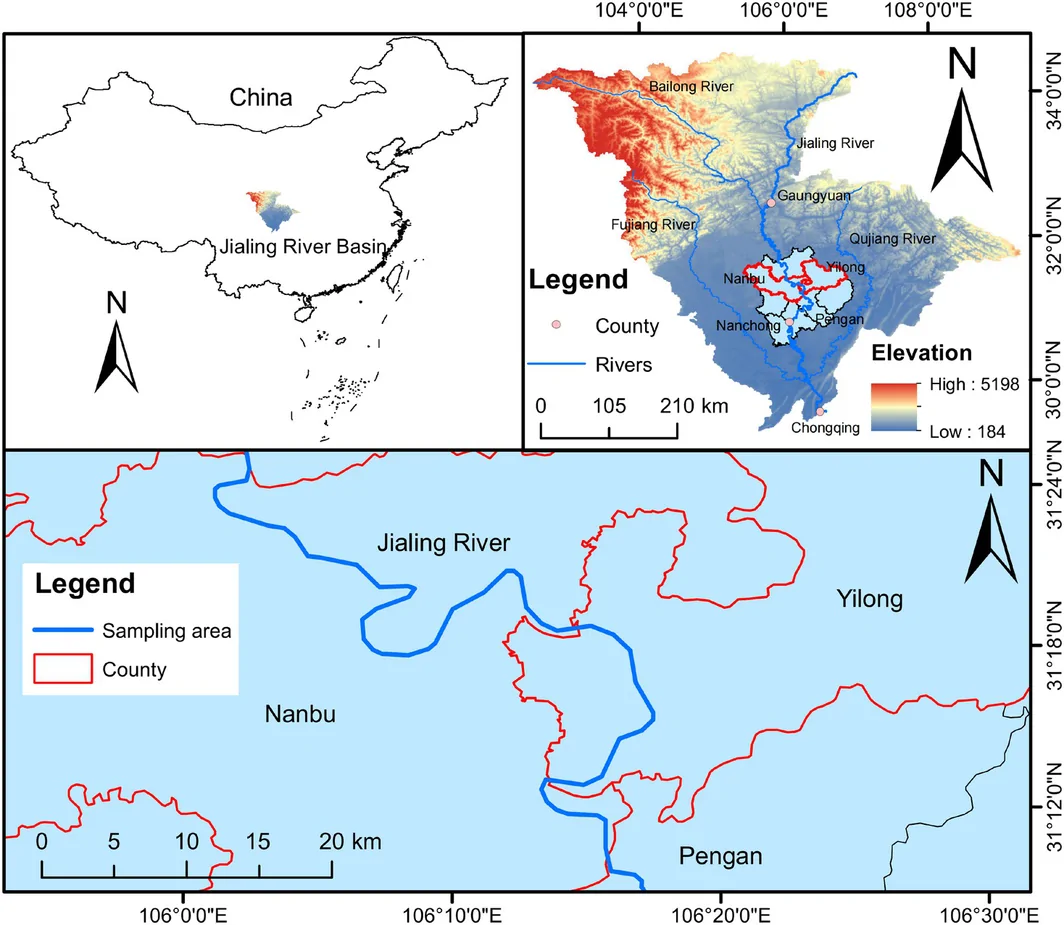
Map of the study area.

Based on this, dominant fish species representing different feeding types were selected from the midstream of the Jialing River between June and July 2024 as research subjects (Qin et al. 2023). These included carnivorous fish: *Hemibagrus macropterus* Bleeker, 1870, and *Siniperca chuatsi* (Basilewsky, 1855); omnivorous fish: *Leiocassis crassilabris* Günther, 1864, *Carassius auratus* (Linnaeus, 1758), *Hemibarbus maculatus* Bleeker, 1871, and *Cyprinus carpio Linnaeus*, 1758; and herbivorous fish: *Xenocypris davidi* Bleeker, 1871, and *Ctenopharyngodon idellus* (Valenciennes, 1844) (Ding 1994; Cao 2024; Chen 1998). This study investigates how dietary differences influence the structure and function of fish gut microbial communities.

Fish intestine collection followed established protocols (Wu et al. 2015; Gajardo et al. 2016). Briefly, individuals of similar body size and without mechanical damage to the body surface were selected. After biological measurements, the body surface was wiped with 75% ethanol. The fish were then immediately dissected to obtain the entire intestinal tract. A longitudinal incision was made along the intestine, and contents from the foregut to the hindgut were collected. The contents were rinsed several times with 0.9% sterile saline. Samples were aliquoted into 2 mL sterile cryovials, rapidly frozen in liquid nitrogen, and transported to the laboratory for storage at −80°C until DNA extraction. All procedures adhered to aseptic techniques to minimize exogenous microbial contamination.

### 2.2 DNA Extraction and 16S rRNA Gene Amplicon Sequencing

The composition of the fish gut microbiota was analyzed using 16S rRNA gene amplicon sequencing. Total genomic DNA was extracted from intestinal content samples using the E.Z.N.A. Soil DNA Kit (Omega Bio‐tek, Norcross, GA, USA). The quality and concentration of the extracted DNA were assessed by 1% agarose gel electrophoresis and a NanoDrop 2000 spectrophotometer (Thermo Fisher Scientific, USA). Qualified DNA samples served as the template for polymerase chain reaction (PCR) amplification of the V3–V4 hypervariable region of the bacterial 16S rRNA gene with the universal primers 338F (5′‐ACTCCTACGGGAGGCAGCAG‐3′) and 806R (5′‐GGACTACHVGGGTWTCTAAT‐3′). The PCR products were purified, and sequencing libraries were constructed using the NEXTFLEX Rapid DNA‐Seq Kit (Bioo Scientific, Austin, TX, USA). Paired‐end sequencing (2 × 250 bp) was subsequently performed on an Illumina NextSeq 2000 (Illumina, San Diego, California, USA).

Raw sequencing reads were first processed with Fastp (v0.19.6) for quality control to remove low‐quality sequences and adapter sequences. Paired‐end reads were then assembled using FLASH (v1.2.11). UPARSE (v7.1) was employed to cluster sequences into operational taxonomic units (OTUs) at a 97% similarity threshold and to remove chimeric sequences. To ensure even sampling depth, all samples were rarefied to the minimum number of sequences per sample. Taxonomic assignment of the OTUs was performed using the RDP classifier (v2.11) against the 16S rRNA gene database (release 138) with a confidence threshold of 70%. All sequencing procedures were conducted by Shanghai Majorbio Bio‐pharm Technology Co. Ltd. The resulting OTU abundance table was subsequently used as the input for functional prediction analyzes.

### 2.3 Functional Prediction Analysis of Gut Microbiota Using PICRUSt2

Functional profiles of the gut microbial communities were predicted based on the operational taxonomic unit (OTU) abundance table derived from 16S rRNA gene sequencing, using PICRUSt2 (Phylogenetic Investigation of Communities by Reconstruction of Unobserved States version 2). This software was used to infer Kyoto Encyclopedia of Genes and Genomes (KEGG) orthologs and calculate the abundances of associated metabolic pathways. To identify KEGG pathways (Level 3) that exhibited significant differences among fish with different feeding habits were identified using the Kruskal–Wallis rank‐sum test was used. The resulting *p*‐values were adjusted for multiple comparisons using the false discovery rate (FDR) correction, with statistical significance defined at an adjusted *p* < 0.05.

### 2.4 Data Processing and Statistical Analysis

Relative abundances of microbial taxa were calculated at both the phylum and genus levels. The top 10 most abundant phyla were selected to generate stacked bar charts and Circos plots (Krzywinski et al. 2009). At the genus level, the top 30 most abundant genera were used to construct heatmaps; the abundance data were normalized using *Z*‐score normalization and visualized by a color gradient.

Microbial alpha diversity within samples was assessed using the Shannon‐Wiener index (for community evenness) (Shannon 1948) and the Chao1 index (for community richness) (Chao 1984), based on the rarefied OTU abundance table. Beta diversity between samples was analyzed using Bray–Curtis dissimilarity and visualized via principal coordinate analysis (PCoA).

LEfSe (linear discriminant analysis effect size) was used to identify differentially abundant taxa across feeding groups, with an LDA score threshold of 3.6. The threshold was set higher than the default of 2.0 to ensure selection of the most specific biomarkers. DESeq2 was additionally applied to carnivorous vs. herbivorous fish for cross‐validation (FDR‐adjusted *p* < 0.05, |log_2_FC| ≥ 1). All differential analyzes were corrected for multiple testing using the Benjamini‐Hochberg method.

Statistical comparisons of alpha diversity indices among groups were conducted using the Kruskal–Wallis test (Kruskal and Wallis 1952), followed by post hoc pairwise Dunn's tests (Dunn 1964). Differences in overall microbial community structure (beta diversity) were tested using permutational multivariate analysis of variance (PERMANOVA) based on Bray‐Curtis distances with 999 permutations. Prior to PERMANOVA, PERMDISP was performed to test for homogeneity of multivariate dispersions among feeding groups. All statistical analyzes and visualizations were performed in R (v4.4.3) using RStudio. Significance levels were denoted as follows: *p < 0.05, **p < 0.01, ***p < 0.001.

## 3 Results

### 3.1 Sample Collection

During the study period, a total of 38 fish specimens were collected, comprising: carnivorous fish (2 species, 12 specimens): *Hemibagrus macropterus* Bleeker, 1870, *Siniperca chuatsi* (Basilewsky, 1855); omnivorous fish (4 species, 19 specimens): *Leiocassis crassilabris* Günther, 1864, *Carassius auratus* (Linnaeus, 1758), *Hemibarbus maculatus* Bleeker, 1871, *Cyprinus carpio* Linnaeus, 1758; and herbivorous fish (2 species, 7 individuals): *Xenocypris davidi* Bleeker, 1871 and *Ctenopharyngodon idellus* (Valenciennes, 1844) (Table 1).

**TABLE 1:** Basic biological information of the eight fish species sampled from the midstream of the Jialing River.

Species	Abbreviation	*N*	Total length (mm)	Weight (g)	Feeding	Habitat
*Siniperca chuatsi* (Basilewsky, 1855)	*S. chu*	7	242.0 ± 21.0	221.7 ± 73.1	Carnivorous	Demersal
*Hemibagrus macropterus* Bleeker, 1870	*H. macro*	5	330.5 ± 64.5	232.7 ± 110.5	Carnivorous	Benthic
*Leiocassis crassilabris* Günther, 1864	*L. cra*	5	266.0 ± 25.0	168.9 ± 50.7	Omnivorous	Benthic
*Hemibarbus maculatus* Bleeker, 1871	*H. mac*	5	317.5 ± 39.5	292.4 ± 95.5	Omnivorous	Demersal
*Carassius auratus* (Linnaeus, 1758)	*C. aur*	5	253.0 ± 62.0	286.1 ± 162.2	Omnivorous	Demersal
*Cyprinus carpio* Linnaeus, 1758	*C. car*	4	525.5 ± 224.5	3143.7 ± 2781.3	Omnivorous	Demersal
*Xenocypris davidi* Bleeker, 1871	*X. dav*	5	361.5 ± 20.5	442.4 ± 79.7	Herbivorous	Demersal
*Ctenopharyngodon idellus* (Valenciennes, 1844)	*C. ide*	2	499.0 ± 35.0	1514.0 ± 359.4	Herbivorous	Demersal

### 3.2 Overview of Sequencing Data

High‐throughput sequencing of the V3–V4 region of the 16S rRNA gene was performed on 38 intestinal samples from the eight fish species. After quality control, 1,074,412 high‐quality sequences were obtained. All samples were rarefied to the minimum sequencing depth of 28,274 sequences per sample to ensure comparability. A total of 5784 operational taxonomic units (OTUs) were identified at a 97% similarity threshold, encompassing multiple microbial phyla.

The rarefaction curves based on the Shannon‐Wiener index reached a stable plateau after approximately 15,000 sequences per sample (Figure 2a), indicating that the sequencing depth was sufficient to capture the majority of microbial diversity and was adequate for downstream analyzes. Venn diagram analysis at the OTU level indicated that only 98 OTUs were shared among all eight fish species, accounting for 1.69% of the total OTUs (Figure 2b), highlighting distinct species‐specific signatures in the gut microbiota. The number of species‐unique OTUs was as follows: *X. dav* (959) > *C. aur* (727) > *H. mac* (329) > *L. cra* (314) > *C. car* (106) > *C. ide* (66) > *H. macro* (37) > *S. chu* (22).

**FIGURE 2:**
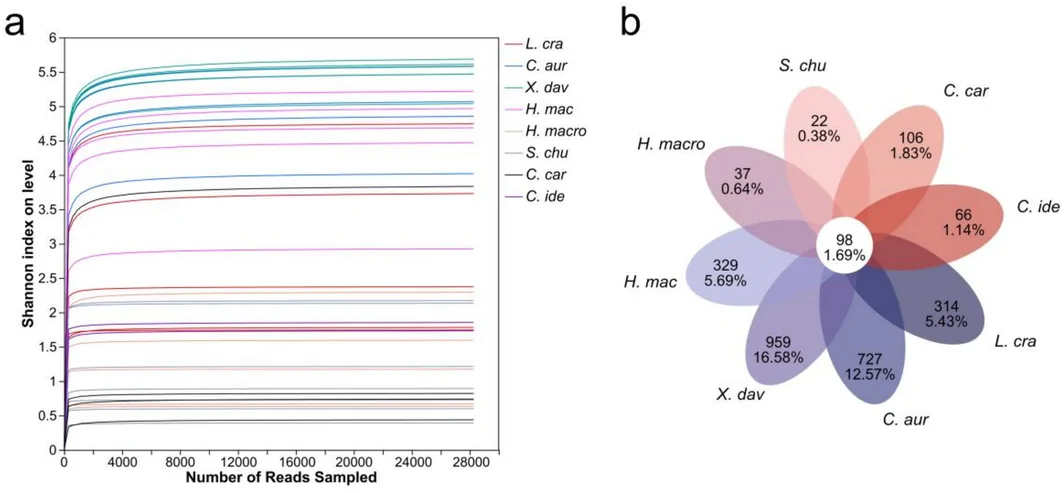
Sequencing overview of the fish gut microbiota. (a) Rarefaction curves based on the Shannon‐Wiener index at the OTU level. The plateau indicates sufficient sequencing depth. (b) Venn diagram at the OTU level showing the distribution of OTUs across the eight fish species. 98 OTUs were shared by all species. The number of unique OTUs per species ranged from 22 (*S. chu*) to 959 (*X. dav*).

### 3.3 Characteristics of Fish Gut Microbiota Composition

#### 3.3.1 Gut Microbiota Structure Across Different Fish Species

Analysis of the gut microbial community composition indicated that the intestinal microbiota of the eight fish species was predominantly composed of the phyla Firmicutes and Proteobacteria. However, significant differences were observed in the relative abundances of these dominant phyla among species (Figure 3a). The relative abundance of Firmicutes was highest in the gut microbiota of *Siniperca chuatsi* , *Hemibagrus macropterus*, and *Cyprinus carpio* (93.46%, 93.34%, and 83.57%, respectively). In contrast, Proteobacteria was the dominant phylum in *Xenocypris davidi* , *Carassius auratus* , and *Hemibarbus maculatus* (45.04%, 35.39%, and 33.62%, respectively), which also showed higher abundances of Actinobacteria (14.19%, 16.11%, and 13.56%, respectively). In the gut microbiota of *Ctenopharyngodon idellus* and *Leiocassis crassilabris* , Firmicutes was the most abundant phylum (61.51% and 71.71%, respectively). Still, Fusobacteria also showed a relatively high abundance (32.75% and 11.72%, respectively).

**FIGURE 3:**
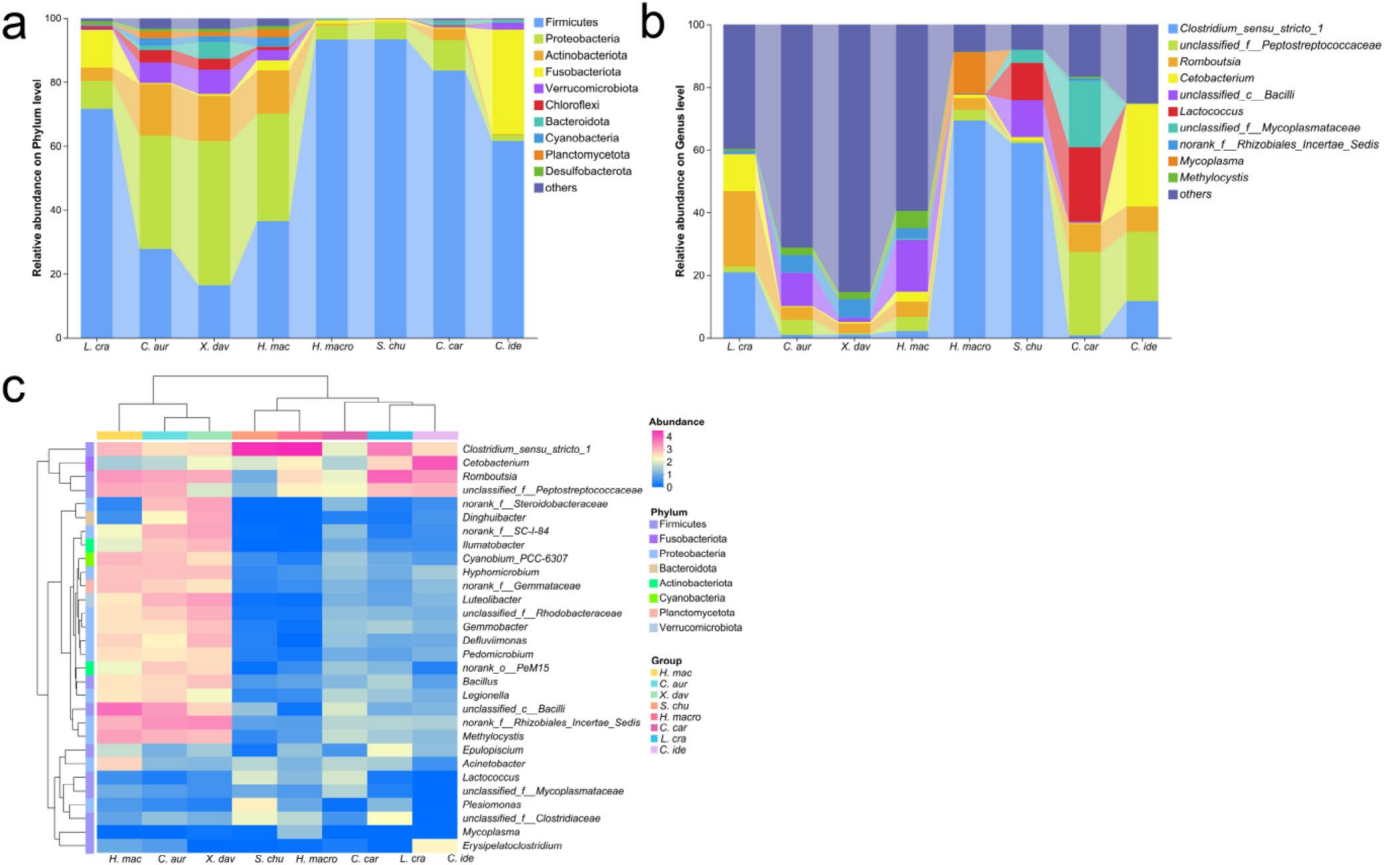
Gut microbial community composition across fish species. (a) Relative abundance at the phylum level (top 10 phyla). (b) Relative abundance at the genus level (top 10 genera). (c) Heatmap of the genus‐level community structure (top 30 genera). The stacked bar charts display the relative abundance distribution based on 16S rRNA gene sequencing (top 10); taxa with a relative abundance below 1% are grouped as “Others.” The heatmap shows the distribution of the top 30 genera across samples. The color scale represents log_10_‐transformed abundance values (0–4), with higher values indicating greater abundance. Pink indicates high abundance, and blue indicates low abundance.

The genus‐level structure further indicated interspecies differences (Figure 3b,c). *Clostridium_sensu_stricto_1* showed relatively high abundances in *Hemibagrus macropterus* and *Siniperca chuatsi* (69.40% and 62.12%, respectively). *Mycoplasma* accounted for 13.12% in *Hemibagrus macropterus*, which was substantially higher than in other fish species (all < 1%). The *Leiocassis crassilabris* was dominated by *Romboutsia* (24.14%), whereas *Cetobacterium* (32.68%) and *unclassified_f_Peptostreptococcaceae* (22.01%) were predominant in *Ctenopharyngodon idellus*. *Cyprinus carpio* was dominated by *unclassified_f_Peptostreptococcaceae* (26.53%) and *Lactococcus* (23.82%). *Unclassified_c_Bacilli* showed relatively high abundances in *Hemibarbus maculatus* and *Carassius auratus* (16.64% and 10.54%, respectively). In contrast, bacterial genera in *Xenocypris davidi* were relatively evenly distributed without a single dominant genus.

#### 3.3.2 Gut Microbiota Structure in Fish With Different Feeding Habits

Analysis of gut microbiota grouped by feeding habit indicated significant differences in community structure at the phylum level (Figure 4a,c). The gut microbiota of carnivorous fish was dominated by the Firmicutes, with relative abundances exceeding 93% in both *Siniperca chuatsi* and *Hemibagrus macropterus*. In contrast, the Proteobacteria showed higher relative abundances in omnivorous (22.43%) and herbivorous (32.72%) fish.

**FIGURE 4:**
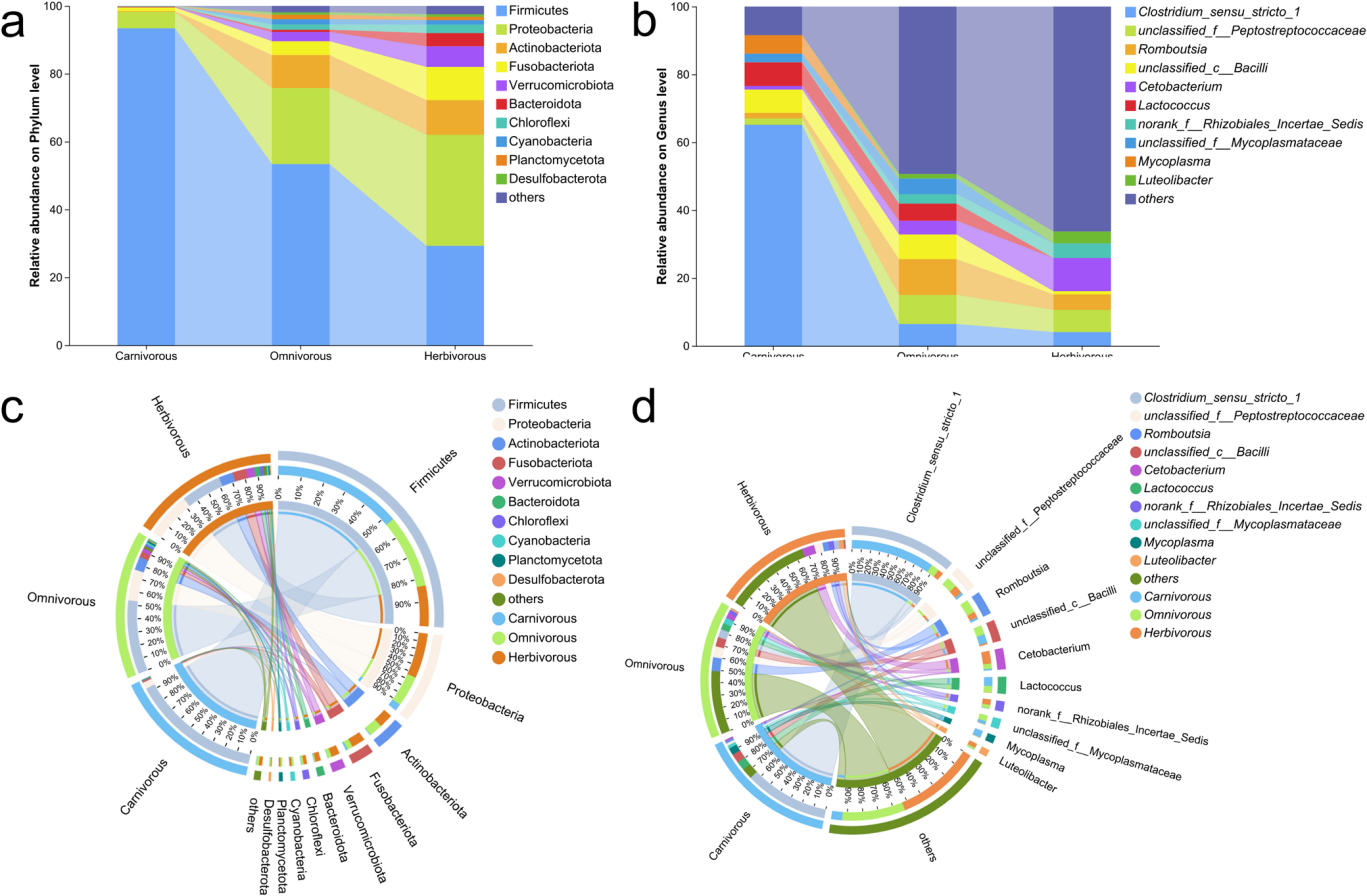
Gut microbial community structure in fish with different feeding habits. (a, b) Relative abundance bar charts at the phylum and genus levels (top 10 genera). (c, d) Circos diagrams at the phylum and genus levels. In the left semicircle, the outer ring represents the feeding groups, and the inner ribbons depict the dominant microbial communities and their relative abundances within each group. In the right semicircle, the outer ring represents the microbial communities, and the inner ribbons show the proportional distribution of each community across feeding groups, with ribbon width corresponding to the distribution proportion. Taxa with a relative abundance below 1% are grouped as “Others.”

Genus‐level analysis further elucidated diet‐associated community patterns (Figure 4b,d). The relative abundance of *Clostridium_sensu_stricto_1* was highest in carnivorous fish but remained below 10% in other feeding groups. Herbivorous fish were dominated by *Cetobacterium* (9.76%), with a relative abundance significantly higher than that in omnivorous (4.06%) and carnivorous (1.05%) fish. Omnivorous fish exhibited greater taxonomic diversity, with *Romboutsia* and *unclassified_f_Peptostreptococcaceae* maintaining high abundances across all omnivorous samples.

### 3.4 Analysis of Gut Microbial Diversity

#### 3.4.1 Diversity Differences Among Feeding Habit Groups

Significant differences in α‐diversity, as measured by the Shannon‐Wiener (*H* = 15.85, *p* = 0.00036) and Chao1 (*H* = 21.23, *p* = 0.00002) indices, were observed among fish with different feeding habits (Figure 5, Kruskal–Wallis). Herbivorous fish exhibited the highest values for both indices, followed by omnivorous fish, while carnivorous fish showed the lowest values (Figure 5a,b). Post hoc Dunn's tests showed significant differences between carnivorous and herbivorous fish, and between omnivorous and carnivorous fish for both indices (Figure 5c,d; *p* < 0.01), while no significant difference was detected between herbivorous and omnivorous fish (*p* > 0.05).

**FIGURE 5:**
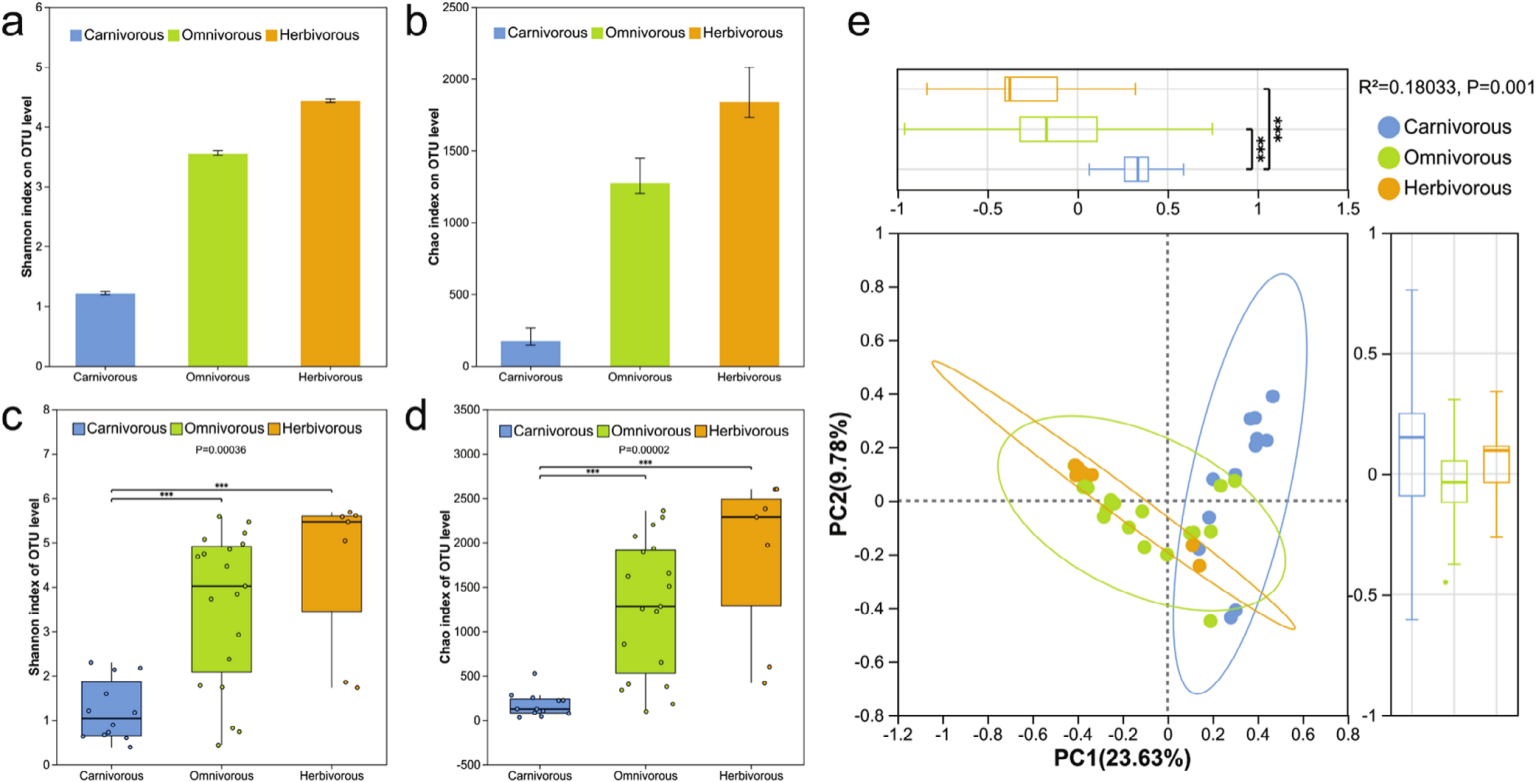
Analysis of α‐ and β‐diversity in the gut microbiota of fish with different feeding habits. (a, b) Distribution of α‐diversity indices: Shannon‐Wiener index (a) and Chao1 index (b). (c, d) Results of post hoc pairwise statistical comparisons for the Shannon‐Wiener (c) and Chao1 (d) indices. (e) β‐diversity visualization: PCoA plot based on Bray‐Curtis distances, with 95% confidence ellipses. In the box plots (a, b), the center line represents the median, the box boundaries indicate the interquartile range (IQR, 25th to 75th percentile), and whiskers extend to 1.5 × IQR. Intergroup differences were assessed using the Kruskal–Wallis test with FDR correction. Significance levels: *p < 0.05, **p < 0.01, ***p < 0.001.

Single‐factor PERMANOVA based on Bray‐Curtis distances indicated that both species identity (Pseudo‐*F* = 3.17, *R* ^2^ = 0.42482, *p* < 0.05) and feeding habit (Pseudo‐*F* = 3.85, *R* ^2^ = 0.18033, *p* < 0.05) significantly influenced gut microbial community structure. PERMDISP analysis indicated homogeneity of multivariate dispersions among feeding groups (*F* = 1.89, *p >* 0.05), validating the PERMANOVA results. Principal coordinates analysis (PCoA) based on Bray–Curtis dissimilarity indicated a significant separation of gut microbial community structure according to feeding habit (Figure 5e). The first two principal coordinates (PC1 and PC2) explained 23.63% and 9.78% of the total community variation, respectively.

### 3.5 Differential Microbial Taxa in Fish Intestines

#### 3.5.1 Analysis of Differential Microbial Taxa in Fish With Different Feeding Habits

Linear discriminant analysis effect size (LEfSe) identified microbial taxa that were differentially abundant among fish with different feeding habits (linear discriminant analysis (LDA) score > 3.6, *p* < 0.05) (Figure 6). The phylogenetic cladogram indicated that herbivorous fish harbored the greatest number of differential taxa (approximately 65%), primarily within Proteobacteria (LDA = 5.17) and Actinobacteriota (LDA = 4.72), including Rhizobiales and Rhodobacterales under the Alphaproteobacteria (Figure 6a). The differential taxa of omnivorous fish were significantly associated with Cyanobacteria (LDA = 3.87) and Planctomycetota (LDA = 3.79). In contrast, the differential taxa for carnivorous fish, although less abundant, showed the highest specificity and were predominantly composed of the Clostridia within Firmicutes (LDA = 5.52) and their corresponding Clostridiales (Figure 6b).

**FIGURE 6:**
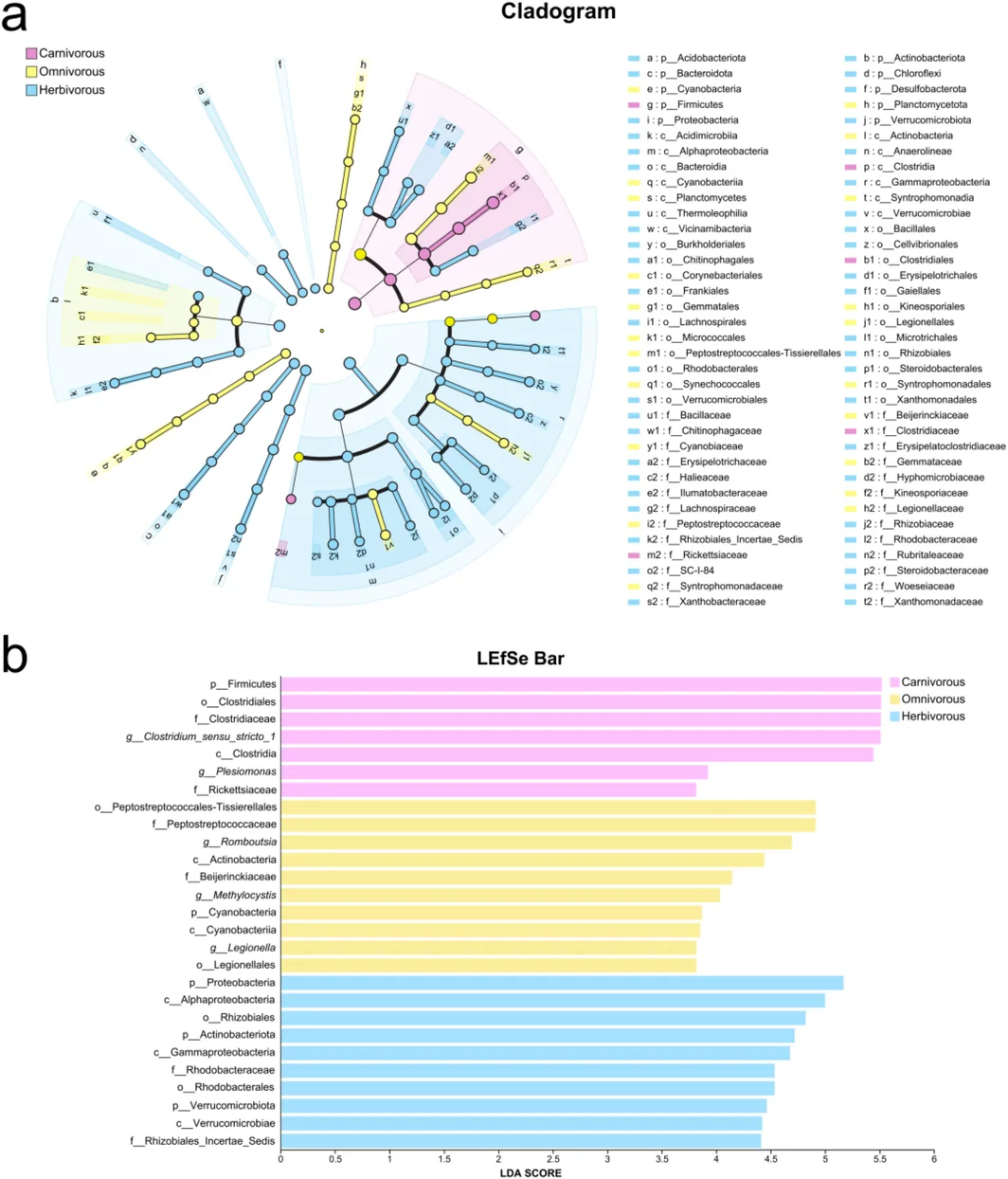
Identification of differentially abundant microbial taxa in fish with different feeding habits. (a) Phylogenetic cladogram of differentially abundant taxa. Nodes are colored according to the feeding group in which the taxon is significantly enriched; light yellow nodes represent taxa with no significant differences among groups. Node size is proportional to the relative abundance of the taxon. (b) Bar chart of linear discriminant analysis (LDA) scores for taxa with an LDA score > 3.6 and statistical significance (Kruskal–Wallis, *p* < 0.05).

DESeq2 was applied to carnivorous vs. herbivorous fish for cross‐validation (FDR‐adjusted *p* < 0.05, |log_2_FC| ≥ 1). A total of 62 significantly differentially abundant OTUs were identified, of which 54 were enriched in carnivorous fish and 8 in herbivorous fish. Taxonomic assignment indicated that OTUs enriched in carnivorous fish were predominantly affiliated with Firmicutes, whereas those enriched in herbivorous fish were mainly assigned to Proteobacteria, consistent with the LEfSe findings.

### 3.6 Functional Prediction Analysis of Gut Microbiota

#### 3.6.1 Characteristics of KEGG Level 3 Functional Profiles

The KEGG Level 3 functional hierarchy provides a detailed classification of specific metabolic pathways, enabling a more precise resolution of functional differences in microbial communities compared to the broader categories of Levels 1 and 2. Therefore, PCoA was performed on the predicted functional profiles of the gut microbiota from fish with different feeding habits, based on KEGG pathway abundances. The results indicated significant differences in predicted functions among groups (PERMANOVA, Pseudo‐*F* = 5.75, *R* ^2^ = 0.24741, *p* < 0.05; Figure 7a). The first three principal coordinate axes explained 75.36% (PC1), 13.87% (PC2), and 4.63% (PC3) of the total variance, respectively. Given the limited intergroup discrimination along PC2 in the graphical representation, PC1 and PC3—which offered higher discriminatory power—were selected for plotting to illustrate the spatial distribution of functional differences more clearly. Together, these two components explain 79.99% of the total variance. Compared to the PCoA results based on taxonomic composition (PERMANOVA, Pseudo‐*F* = 3.85, *R* ^2^ = 0.18033, *p* < 0.05; Figure 5e), the functional profiles indicated greater explanatory power for the observed intergroup variation.

**FIGURE 7:**
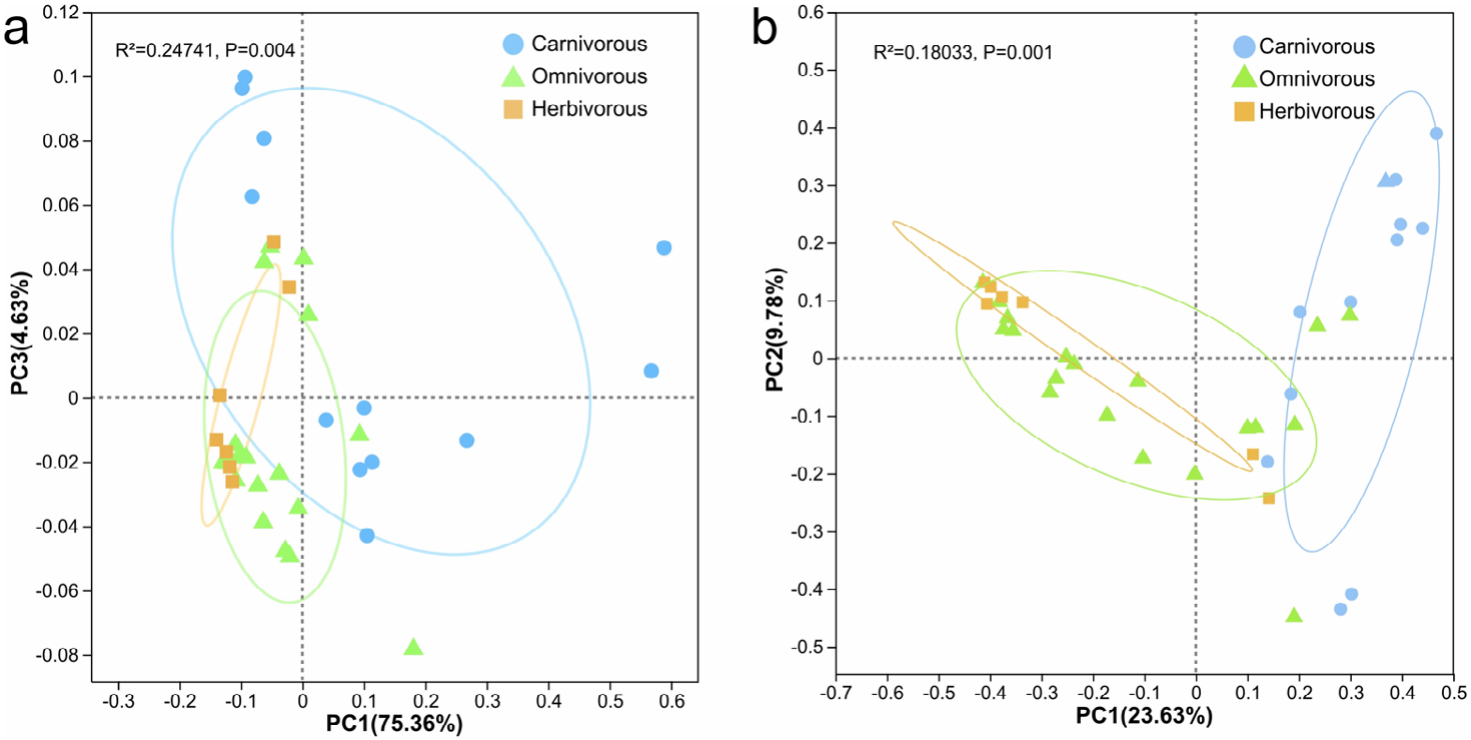
Comparison of gut microbial functional profiles and taxonomic composition β‐diversity among fish with different feeding habits. (a) PCoA plot (Bray‐Curtis distance) based on KEGG Level 3 pathway abundance, revealing differences in the predicted functional profiles of gut microbiota among fish with different feeding habits. (b) PCoA analysis of taxonomic composition (reproducing Figure 5e) indicates differences in gut microbial community structure among fish with different feeding habits. Confidence ellipses represent 95% confidence intervals. Coordinate axes are labeled with the percentage of variance explained.

#### 3.6.2 Significantly Enriched KEGG Level 3 Pathways

Functional prediction based on PICRUSt2 indicated significant differences in the abundance of KEGG Level 3 pathways among the gut microbiota of fish with different feeding habits (Figure 8). Carnivorous fish exhibited higher relative abundances in pathways including starch and sucrose metabolism, the phosphotransferase system (PTS), and cysteine and methionine metabolism. Omnivorous fish showed moderate abundance levels across multiple metabolic pathways. The pathways for microbial metabolism in diverse environments and quorum sensing displayed abundances intermediate between those of carnivorous and herbivorous fish. Herbivorous fish exhibited the highest relative abundances in pathways such as carbon fixation pathways in prokaryotes, oxidative phosphorylation, and glyoxylate and dicarboxylate metabolism.

**FIGURE 8:**
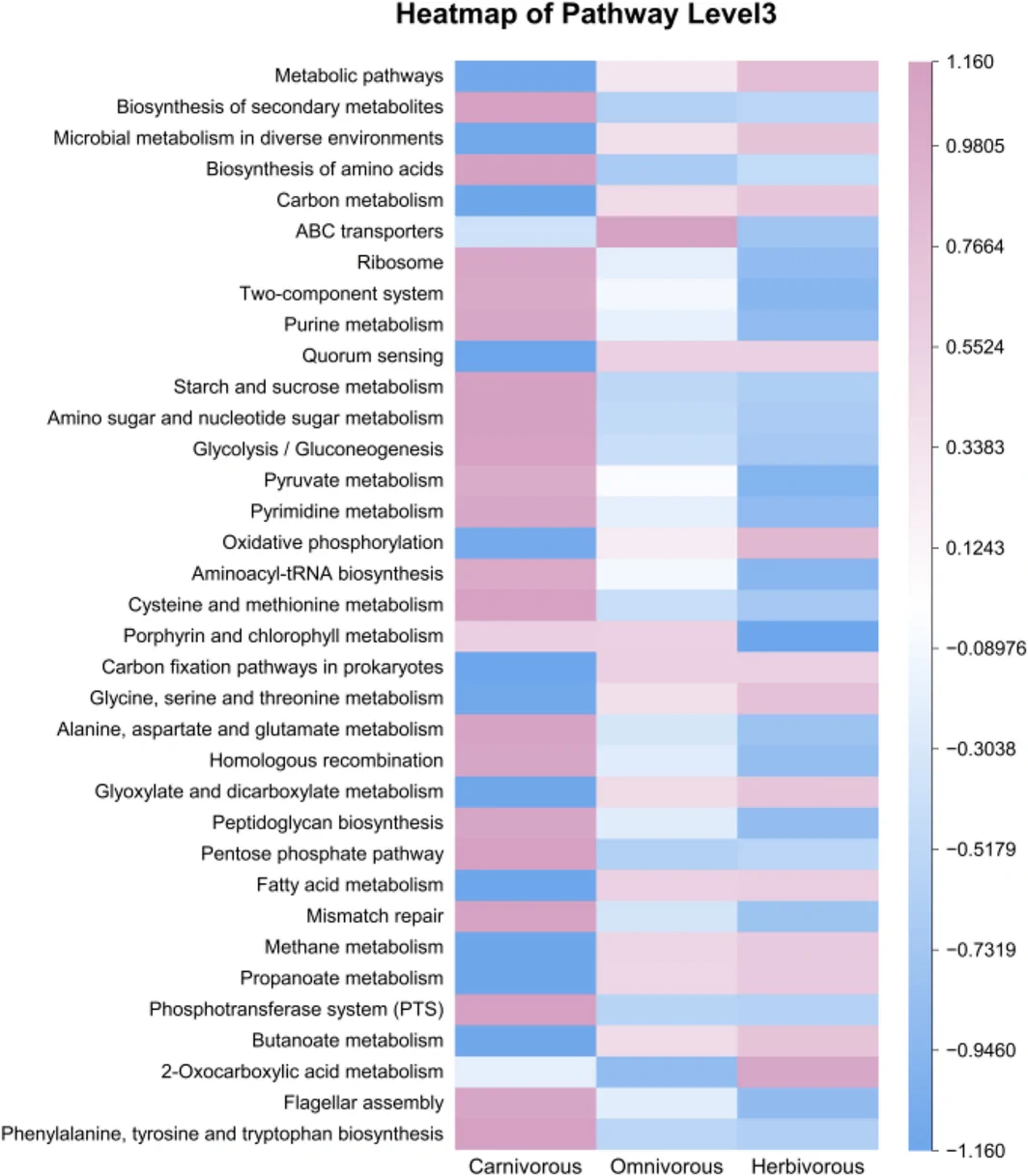
Heatmap of KEGG Level 3 pathway relative abundances in the gut microbiota of fish with different feeding habits. Only the top 35 pathways (ranked by average relative abundance across the three feeding groups) are shown. The color scale represents row‐wise *Z*‐score standardized values, with pink indicating higher abundance and blue indicating lower abundance.

#### 3.6.3 Intergroup Differential Analysis of Key KEGG Level 3 Pathways

To systematically characterize the functional features of gut microbiota in fish with different feeding habits, five representative metabolic pathways were screened based on intergroup differential analysis of KEGG Level 3 pathway profiles (Kruskal–Wallis rank‐sum test, FDR‐corrected, with Dunn's post hoc comparison). Among these, three pathways characterize core metabolic features associated with specific feeding habits, while two pathways showed unexpected enrichment in carnivorous fish (Figure 9). Regarding pathways characterizing diet‐specific metabolic functions: the cysteine and methionine metabolism pathway exhibited the highest relative abundance in carnivorous fish (1.026% ± 0.051%), reflecting an adaptation to high‐protein diets and the consequent elevated metabolic demand. The microbial metabolism in diverse environments pathway was significantly enriched in omnivorous fish (4.792% ± 0.221%), with its abundance being significantly higher than in the carnivorous group (*p* < 0.05), which aligns with their dietary diversity. The carbon fixation pathways in prokaryotes pathway showed the highest abundance in herbivorous fish (0.927% ± 0.091%) and was significantly higher than in the carnivorous group (0.781%, *p* < 0.05), indicating an adaptation to a diet primarily composed of plant material.

**FIGURE 9:**
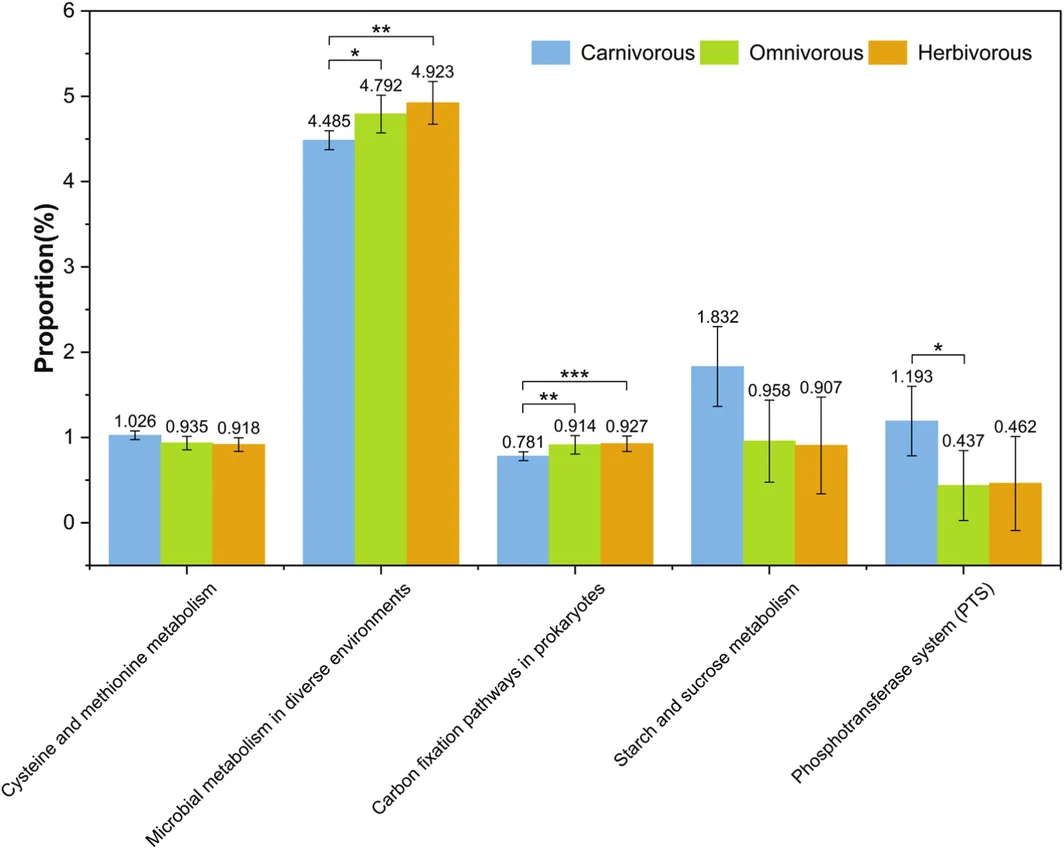
Relative abundance (%) of KEGG Level 3 metabolic pathways showing significant differences between dietary groups. Data are presented as mean ± standard deviation, with numerical labels above bars indicating mean values. Intergroup differences were assessed using the Kruskal–Wallis test (FDR‐corrected) followed by Dunn's post hoc test (95% confidence intervals). Significance levels: *p < 0.05, **p < 0.01, ***p < 0.001.

In carnivorous fish, the relative abundances of both the starch and sucrose metabolism pathway and the phosphotransferase system (PTS) pathway were the highest (1.832% ± 0.467% and 1.193% ± 0.408%, respectively). This finding contrasts with the traditional view that carnivorous fish possess a limited capacity for carbohydrate utilization. These results indicate that, despite the low carbohydrate content in the diet of carnivorous fish, their gut microbial communities retain metabolic pathways for degrading and utilizing such substrates. This suggests that under high‐protein, low‐carbohydrate dietary conditions, carnivorous fish may maintain corresponding carbohydrate metabolism in their gut microbiota to adapt to potentially unstable sugar availability from prey.

## 4 Discussion

### 4.1 Association Between Feeding Habits and the Structure of Fish Gut Microbiota

This study analyzed the gut microbiota of fish with different feeding habits, indicating that diet is an important factor influencing gut microbial community structure. Analysis at the phylum level indicated significant differences among fish with different diets. The gut microbiota of carnivorous fish was dominated by the Firmicutes (> 93%), a compositional feature similar to that reported in carnivorous mammalian species, which may be an related to their high‐protein, high‐fat diet (Wu, Pan, et al. 2024; Wu, Xiao, et al. 2024; Louvado et al. 2024). As primary carbohydrate fermenters, Firmicutes produce substantial amounts of short‐chain fatty acids (SCFAs) (Rimoldi et al. 2023), which play crucial roles in maintaining intestinal barrier integrity, regulating immune cell function, and suppressing inflammatory responses, thereby enhancing host antioxidant capacity and immune homeostasis (Fontinha et al. 2024). In contrast, herbivorous and omnivorous fish exhibited higher relative abundances of Proteobacteria compared to carnivorous fish, resulting in a more diverse composition at the phylum level. Studies indicate that Proteobacteria can utilize diverse organic compounds as carbon and nitrogen sources (Wu et al. 2025), providing various functions related to biogeochemical cycles that may support host adaptation to different environmental conditions (Zeng et al. 2021; Tolas et al. 2025). These differences in community composition are likely linked to variations in the chemical composition of the diets consumed by fish with different feeding habits.

Analysis at the genus level further indicated signature microbial taxa closely associated with specific feeding habits. *Clostridium_sensu_stricto_1*, which was significantly enriched in carnivorous fish, is a common protein‐fermenting bacterium. Its high abundance likely represents an adaptation to the host's nitrogen metabolism demands under a high‐protein diet. This genus can also produce SCFAs like butyrate through carbohydrate fermentation, which may help optimize the intestinal microenvironment and maintain host gut health (Fang et al. 2023). *Cetobacterium*, specifically enriched in herbivorous fish, has been shown to synthesize vitamin B12 in the intestines of freshwater fish (Zhu et al. 2025; Qi et al. 2023). Since plant‐based diets are typically deficient in this vitamin, the high abundance of *Cetobacterium* may indicate a reliance of herbivorous fish on their gut microbiota for vitamin B12 synthesis, which is crucial for energy metabolism and normal growth and development. Furthermore, LEfSe identified several microbial taxa in herbivorous fish that originate from terrestrial environments, such as Rhizobiales and Rhodobacterales, suggesting potential cross‐ecosystem microbial transfer mediated by feeding behavior (Erlacher et al. 2015). In contrast, omnivorous fish exhibited a more diverse genus‐level composition, with *Romboutsia* and *unclassified_f__Peptostreptococcaceae* maintaining high abundance across all omnivorous samples (Liu et al. 2023). This relatively balanced and diverse composition suggests, from an ecological perspective, more versatile metabolic functions, which may enable omnivorous fish to adapt more flexibly to environmental changes.

In summary, this study further indicates, from the perspective of fish gut microbiota, that feeding habit is a key factor influencing gut microbial community structure, and the gut microbiota may serve as a potential bioindicator reflecting the nutritional health status of fish. Therefore, monitoring the dynamic changes in the gut microbiota of specific fish provides an important theoretical basis for assessing the quality of their habitat environment and for implementing early warning systems and targeted conservation strategies for fish resources.

### 4.2 Effects of Feeding Habits on Gut Microbial Diversity in Fish

The diversity characteristics of fish gut microbial communities serve as crucial indicators for evaluating their ecological functions and environmental adaptability. This study indicated that herbivorous fish exhibited the highest Shannon‐Wiener index (mean 4.42) and Chao1 index (mean 1836.7), followed by omnivorous fish, while carnivorous fish showed the lowest values for both indices (Shannon‐Wiener: 1.21, Chao1: 173.99). This disparity correlates closely with the distinct trophic niches occupied by different feeding types. The diets of herbivorous fish are rich in structural carbohydrates such as cellulose and hemicellulose, the degradation of which relies on synergistic metabolism within complex gut microbial communities (Liu et al. 2023). This functional requirement ecologically supports higher species richness and evenness. In contrast, the diets of carnivorous fish are dominated by proteins and lipids (Wu, Pan, et al. 2024; Wu, Xiao, et al. 2024; Jiao et al. 2023). These substrates can be efficiently utilized through relatively specialized metabolic pathways (e.g., protein fermentation and fatty acid β‐oxidation), often dominated by anaerobic taxa such as the class Clostridia within the phylum Firmicutes, resulting in a community structure characterized by lower taxonomic diversity but higher functional specificity.

At the community β‐diversity level, single‐factor PERMANOVA based on Bray‐Curtis distances showed that feeding habit explained 18.03% of the community structural variation (*R* ^2^ = 0.18033, *p* < 0.05), indicating that feeding habit is an important factor influencing community structure. Species identity also showed a significant effect (*R* ^2^ = 0.42482, *p* < 0.05), suggesting that other ecological factors, including host species and water physicochemical parameters, collectively contribute to this process (Lin et al. 2025). PERMDISP analysis (*p* > 0.05) indicated the validity of the PERMANOVA assumptions. In the PCoA plot, samples from carnivorous fish exhibited the highest degree of clustering, indicating that their gut microbial structure is more strongly influenced by feeding habit compared to other groups and that their communities show lower sensitivity to external environmental fluctuations. In contrast, samples from herbivorous and omnivorous fish were more dispersed, suggesting their gut microbiota may be more sensitive to subtle variations in dietary composition or environmental disturbances.

In summary, this study further indicates from a diversity perspective that diet is a key factor influencing the assembly of fish gut microbial communities. The findings indicate that carnivorous fish, typically occupying higher trophic levels, exhibit lower gut microbial diversity, which is likely an adaptation to their efficient energy acquisition strategy. Conversely, herbivorous fish, generally residing at lower trophic levels, maintain highly diverse gut microbial communities to accommodate the complex composition of their plant‐based diets. These findings provide a theoretical basis for elucidating the mechanisms by which diet influences the formation of fish gut microbiota. They also suggest that fish gut microbial communities could serve as potential bioindicators for assessing aquatic environmental quality and food web health, offering scientific insights for the conservation of aquatic ecosystems and the sustainable management of fish resources.

### 4.3 Metabolic Characteristics of Fish Gut Microbiota Based on PICRUSt2 Functional Prediction

Functional prediction analysis of the gut microbiota using PICRUSt2 indicated that feeding habit is an important factor influencing the functional profiles of fish gut microbiota (PERMANOVA, *R* ^2^ = 0.24741, *p* < 0.05), with a higher explanatory power than that of taxonomic composition (*R* ^2^ = 0.18033). This suggests that fish with different diets may perform similar ecological functions through distinct gut microbial communities, reflecting functional convergence at the metabolic level. In‐depth analysis of KEGG Level 3 pathways further elucidated diet‐related metabolic adaptation strategies.

The enrichment of the cysteine and methionine metabolism pathway in the gut microbiota of carnivorous fish aligns with their demand for sulfur amino acid metabolism under a high‐protein diet (Zhou et al. 2021). This pathway plays a central role in protein synthesis, the supply of methyl donors (S‐adenosylmethionine), and the production of the antioxidant molecule glutathione (Martins et al. 2021). The highest relative abundances in the gut microbiota of carnivorous fish were observed for the starch and sucrose metabolism and phosphotransferase system (PTS) pathways. This finding contrasts with the conventional view that carnivorous fish possess a limited capacity for carbohydrate utilization (You et al. 2020). The PTS is a specialized transport system enabling bacteria to efficiently uptake and phosphorylate various monosaccharides (Jeckelmann and Erni 2020). Its high abundance suggests that the microbiota has an efficient competitive uptake capacity for trace fermentable carbon sources in the environment. Together with the starch and sucrose metabolism pathway, this finding indicates that within the high‐protein, low‐carbohydrate gut environment of carnivorous fish, their microbial communities retain the metabolic capacity to process potential glycogen or structural carbohydrates present in prey. Within the ecological context of habitat fragmentation caused by cascade channelization in the Jialing River, the retention of such metabolic functions may provide advantages in coping with spatiotemporal instability in natural prey resources.

The enrichment of pathways such as carbon fixation pathways in prokaryotes and glyoxylate and dicarboxylate metabolism in the gut microbiota of herbivorous fish may represent functional patterns for degrading and fermenting complex carbohydrates in plant‐based diets. Among these, the glyoxylate cycle—the core function of the glyoxylate and dicarboxylate metabolism pathway (Huang et al. 2023)—effectively replenishes precursors required for biosynthesis by bypassing the decarboxylation steps of the tricarboxylic acid (TCA) cycle (Zhou et al. 2023). Concurrently, carbon fixation pathways may assist microbes in refixing and reutilizing the high concentrations of fermentation‐derived CO_2_ within the gut (Ju et al. 2023). The synergy of these two mechanisms may optimize the carbon metabolic network efficiency of the gut microbiota under a fiber‐dominant diet. In the gut microbiota of omnivorous fish, the significant enrichment of the microbial metabolism in diverse environments pathway reflects the high plasticity of their metabolic networks. This pathway integrates multiple alternative pathways for carbon source utilization, nitrogen transformation, and energy metabolism, enabling a flexible response to the dynamic changes in diverse nutritional substrates—including proteins, carbohydrates, and lipids—within an omnivorous diet (Sturtz et al. 2026; Coutinho et al. 2016). This multifunctional metabolic trait may represent a key microbial adaptation mechanism for omnivorous fish to maintain energetic homeostasis during seasonal transitions or habitat disturbances.

### 4.4 Implications for the Protection of Fish Gut Microbiota in Channelized Habitats

This study analyzed the community structure and functional characteristics of gut microbiota in fish with different feeding habits within the cascade‐channelized section of the Jialing River. The gut microbiota of carnivorous fish was dominated by the Firmicutes and the *Clostridium_sensu_stricto_1*. Herbivorous fish showed significant enrichment of the Proteobacteria and the *Cetobacterium*. Omnivorous fish exhibited higher genus‐level diversity. Feeding habit significantly influenced the structure of fish gut microbial communities (PERMANOVA, Pseudo‐*F* = 3.85, *R* ^2^ = 0.18033, *p* < 0.05). The α‐diversity of gut microbes in herbivorous fish was significantly higher than in carnivorous fish (*p* < 0.05). This finding aligns with studies conducted in naturally flowing rivers and lakes, reflecting universal patterns in gut microbial communities across different feeding strategies. Functionally, the associations “carnivorous – cysteine and methionine metabolism,” “herbivorous – carbon fixation pathways in prokaryotes,” and “omnivorous – microbial metabolism in diverse environments” were consistent with the functional profiles of gut microbiota in fish of corresponding feeding habits in natural waters. This indicates that core feeding habit‐function linkages are preserved even in channelized environments.

However, contrary to conventional understanding, the gut microbiota of carnivorous fish in this study exhibited higher relative abundances in the starch and sucrose metabolism and the phosphotransferase system (PTS) pathways. This phenomenon differs from findings in most natural flowing rivers and lakes. Cascade dams reduce flow velocity and shift habitats toward lentic conditions. Previous studies have shown that cascade development can substantially alter fish community structure (Wang et al. 2021) and, through food web cascade effects, transform benthic macroinvertebrate communities from lotic‐adapted to lentic‐adapted species. Environmental factors have a significant influence on fish gut microbial community structure (Singh et al. 2025). Lentic habitats may support more abundant benthic invertebrates (e.g., chironomid larvae), which are rich in glycogen. Benthic invertebrates are capable of assimilating and metabolizing energy‐rich organic matter (White et al. 2017), making them a potentially energy‐rich prey for carnivorous fish. The enrichment of carbohydrate metabolism pathways in carnivorous fish gut microbiota might be associated with alterations in prey communities following channelization, although this speculation requires further investigation.

Although the core diet‐microbiota association pattern persists, ecological management of reservoirs should consider restoring the diversity and stability of riverine prey communities by regulating hydrological heterogeneity, which is crucial for maintaining the healthy expression of fish gut microbial ecological functions. This study provides baseline data for understanding the conservation of fish gut microbiota in channelized rivers and offers a reference for developing microbiota‐based conservation strategies.

### 4.5 Limitations and Future Perspectives

This study presents correlative and predictive findings regarding the gut microbiota of fish in a channelized river system. The functional interpretations are based on PICRUSt2 predictions rather than direct measurements of gene expression or metabolic activity. The accuracy of PICRUSt2 is constrained by the completeness of reference genome databases, particularly for non‐model aquatic organisms and taxa such as Fusobacteria and *Cetobacterium*, whose functional repertoires remain poorly characterized in public databases. The inferred functional profiles therefore represent metabolic potential rather than actual pathway activity.

The study design lacks a non‐channelized control system, which limits the ability to attribute observed patterns specifically to cascade channelization. The findings describe diet‐related patterns within the channelized system rather than demonstrating channelization‐driven changes. In addition, sample sizes were uneven across feeding groups, with *Ctenopharyngodon idellus* limited to two individuals compared to four to seven individuals for other species. This restricts the representativeness of the gut microbiota for this species and may affect statistical power and the reliability of group‐level comparisons, including PERMANOVA.

Future investigations would benefit from integrating metatranscriptomics and metabolomics to verify whether the predicted functional pathways are actively expressed. Stable isotope tracing could help elucidate how habitat alterations influence food web structure and subsequently affect gut microbial functional profiles. Seasonal sampling would further clarify temporal dynamics of gut microbial communities in response to environmental fluctuations. Expanding sample sizes for underrepresented species would also strengthen the robustness of statistical comparisons. These approaches would improve the understanding of causal relationships between habitat modification and fish gut microbiota functions.

## 5 Conclusion

This study indicates that both feeding habit and host species identity significantly influence gut microbial community structure in fish from the channelized Jialing River, with species identity explaining a larger proportion of the variation. Carnivorous fish were characterized by enrichment of amino acid metabolism pathways, herbivorous fish by carbon fixation pathways, and omnivorous fish by diverse environmental metabolism pathways, and these diet‐related patterns were preserved in the channelized system. Notably, the enrichment of carbohydrate metabolism pathways (starch and sucrose metabolism, phosphotransferase system) in carnivorous fish was observed in the channelized river, suggesting a potential link to prey community alterations following channelization, although this requires further verification with non‐channelized controls.

These findings provide a theoretical basis for using fish gut microbiota as a bioindicator of dietary and habitat conditions, and also offer a theoretical foundation for the conservation of fish resources and aquatic ecological environments in channelized rivers. From a management perspective, maintaining hydrological heterogeneity and prey community diversity in reservoir operations is recommended to support the ecological functions of fish gut microbiota. Future studies incorporating metatranscriptomics, metabolomics, and seasonal sampling are needed to validate the functional activity of these pathways and to disentangle the effects of feeding habit from those of host phylogeny and environmental change.

## Author Contributions

**Mengying Hu:** conceptualization (lead), data curation (lead), methodology (equal), software (lead), writing – original draft (lead). **Chang Chen:** conceptualization (equal), investigation (equal). **Yuan Feng:** methodology (equal), validation (equal). **Liang Liu:** investigation (equal), methodology (equal). **Xiaopeng Tang:** investigation (equal), validation (equal). **Heran Wang:** investigation (equal), validation (equal). **Fubin Zhang:** conceptualization (equal), funding acquisition (lead), investigation (equal), writing – review and editing (lead).

## Funding

This work was supported by the National Natural Science Foundation of China, 31901219. Education Department of Sichuan Province through the Sichuan Provincial Innovation and Entrepreneurship Training Program for College Students (Grant No. S202510638078). Natural Science Foundation of Sichuan Province (2026YFTX0020).

## Conflicts of Interest

The authors declare no conflicts of interest.

## Data Availability

The data that support the findings of this study are available from the corresponding author upon reasonable request. All data generated or analyzed in this study are contained in the Science Data Bank (https://www.scidb.cn/s/mMfEj2) and the NCBI Sequence Read Archive under BioProject accession number [PRJNA1504619] (http://www.ncbi.nlm.nih.gov/bioproject/1504619).
